# Physical activity and mental health trends in Korean adolescents: Analyzing the impact of the COVID-19 pandemic from 2018 to 2022

**DOI:** 10.1515/med-2024-0978

**Published:** 2024-07-10

**Authors:** Kyungsik Kim, Zixiang Zhou, Xiao Ren, Xiuxiu Bu, Xiaodai Jia, Qingyang Shao

**Affiliations:** Department of Sport & Leisure Studies, Hoseo University, Asan-si, Republic of Korea; School of Physical Education, Hunan University of Science and Technology, Xiangtan, 411100, China; Department of Physical Education, Hunan Agricultural University, Changsha, China; Department of Physical Education, Qiannan Normal University for Nationalities, Duyun, China

**Keywords:** adolescents, COVID-19, depression, mental health, physical activity, stress

## Abstract

**Objective:**

Mental health significantly affects the physical and emotional development of adolescents. The aim of the current study was to examine how physical activity (PA) and mental health among Korean adolescents changed before and after the start of the COVID-19 pandemic.

**Methods:**

We used comparative cross-sectional methods, gathering information from the Korea Disease Control and Prevention Agency databases and conducting Chi-square testing and correlation analysis for evaluation.

**Results:**

The findings indicate that before and during the pandemic, participation in both moderate- and high-intensity PA, as well as strength training, mitigated stress, depression, and suicidal ideation. Furthermore, the findings confirm the beneficial effects of various physical activities on mental well-being.

**Conclusions:**

These insights emphasize the vital role of regular PA in improving mental health among adolescents, particularly during a health crisis such as the pandemic.

## 1 Introduction

The COVID-19 pandemic has had a vast global impact, with over 760 million cases, marking its place as a significant event in history. COVID-19 continues to pose threats to the health of adolescents and young people [1]. To combat its spread, governments implemented various temporary measures during high-risk phases of the pandemic, such as community quarantines, school closures, and activity restrictions [2]. These interventions significantly altered the daily lives of many adolescents, with notable re-percussions on mental health [3]. In January 2022, the Centers for Disease Control and Prevention released data highlighting the mental health risks posed by the pandemic to adolescents. Their analysis shows that 37% of high school students reported poor mental health and that 44% felt sad or hopeless in the preceding year [1]. Concurrently, multiple findings underscore the decline in physical performance among adolescents, likely due to reduced physical activity (PA) during the pandemic [4–7]. These data indicate that both the physical and mental well-being of adolescents faced unparalleled challenges during the COVID-19 outbreak.

Periods of heightened COVID-19 risk have had an impact on several aspects of adolescents’ daily lives. Restrictions on activities in community and public places, for example, decreased outdoor PA among this group [8]. Furthermore, measures to contain the outbreak had adverse psychological consequences for adolescents. Factors include stress from social distancing policies, academic pressures stemming from subpar distance learning experiences, and heightened anxiety from extensive outbreak information campaigns [9–11].

Contextualizing these findings within a broader perspective is crucial. While numerous findings highlight the negative impact of the pandemic on adolescent mental health [12,13], the prevalence of mental health issues among adolescents differs significantly across countries [14]. For instance, findings from a multi-country cross-sectional study by Nguyen et al. [15] demonstrate varying correlations between the impact of the pandemic and adolescent stress. The correlations were positive and moderate in Morocco and Serbia, positive and weak in Vietnam and the United States, and negative and weak in Sweden [15]. These differences suggest that the effects of COVID-19 on adolescents, particularly concerning stress, depend on their respective locales.

Although many scholars have presented findings on the association between COVID-19 and Korean adolescent mental health (e.g., stress, depression, and suicidal ideation) and PA, these studies have important limitations. Some scholars analyzed the mental health of Korean adolescents during the COVID-19 pandemic and summarized positive findings in comparison to the past, as reflected in decreases in subjective stress, depression, and suicide [16–19]. However, other findings indicate an increase in the prevalence of depression and anxiety among Korean adolescents during the COVID-19 pandemic, as well as an increase in the rate of medical visits due to mental illness [20]. These inconsistencies suggest that scholars need to consider other factors in mental health changes. To this end, some scholars have analyzed the association between PA and mental health among Korean adolescents. The findings show that PA positively influenced mental health [21] and that higher levels of PA were related to lower COVID-19 stress [22]. Various findings confirm that PA had a negative impact on stress and that groups participating in moderate PA typically had lower stress levels [23,24]. In terms of depression, some findings show that PA interventions reduced depressive symptoms in adolescents [25,26]. Previous findings also show that increasing PA is an effective way to prevent suicide [27,28].

A correlation also exists between reduced PA, deteriorating mental health, and confinement due to infectious diseases [8,29,30]. However, these studies have significant limitations. Notably, many scholars did not conduct comparative analysis of periods before and during the COVID-19 pandemic and often did not use national data in their sampling. Without such pre-pandemic comparisons, ascertaining whether observed changes in mental health and PA directly result from the pandemic or depend on other external factors is difficult. Moreover, the absence of nationwide data in many of these studies hampers the generalizability of the findings. Regional variations and cultural differences can profoundly influence the relationship between PA and mental health. To address these shortcomings, we used nationwide data sourced from the Korea Centers for Disease Control and Prevention (KCDC). By comparing data from before and during the COVID-19 pandemic, we sought a holistic understanding of how the pandemic affected the mental health and PA of Korean adolescents.

PA plays a pivotal role in sustaining physical health and fostering mental development. However, epidemic-related restrictions can curtail opportunities for such activity, thereby challenging both physical and mental well-being [31]. Reduced PA can result in a decline in mental health, manifesting as symptoms of anxiety, depression, and stress, which in turn increase the risk of more severe mental illnesses [32]. Given these relationships, examining alterations in PA and mental health among adolescents can help policymakers tailor health promotion and PA initiatives to the needs of adolescents. Resulting interventions would not only benefit their immediate well-being but also strengthen overall epidemic response strategy. Hence, delving into the shifts in PA and mental health among Korean adolescents during the COVID-19 pandemic can promote holistic health and well-being. To address the shortcomings of previous studies, we identified changes in and the relationship between PA and mental health among adolescents before and during the COVID-19 pandemic using nationwide data. We proposed the following hypotheses:

H1. PA level before and during the COVID-19 pandemic will differ.

H2. Adolescents participating in moderate- and high-intensity PA and strength training will experience lower stress levels than those who do not participate in such activities.

H3. Adolescents participating in moderate- and high-intensity PA and strength training will experience lower depression levels than those who do not participate in such activities.

H4. Adolescents participating in moderate- and high-intensity PA and strength training will experience lower suicidal ideation levels than those who do not participate in such activities.

H5. Correlations will emerge among pandemic, age, PA, and mental health for adolescents.

## 2 Methods

### 2.1 Study design and data collection

We used raw data from the annual Adolescent Health Behavior Survey conducted by the KCDC, which provides survey data to support research on adolescent health. We used this data in accordance with KCDC guidelines. We adopted a comparative cross-sectional approach to assess shifts in PA and mental health among Korean adolescents, analyzing data spanning several intervals. Comparative cross-sectional analysis considers different populations or groups within those populations to identify changes in the prevalence or distribution of specific characteristics or conditions. This type of research helps researchers understand the differences and similarities between groups and explore factors that influence these disparities. We divided the timeline into two periods: pre-pandemic (2018–2019) and pandemic (2020–2022). Due to KCDC’s practice of sampling new research subjects annually, the data used to compare the two periods did not come from the same subjects.

**Figure 1:**
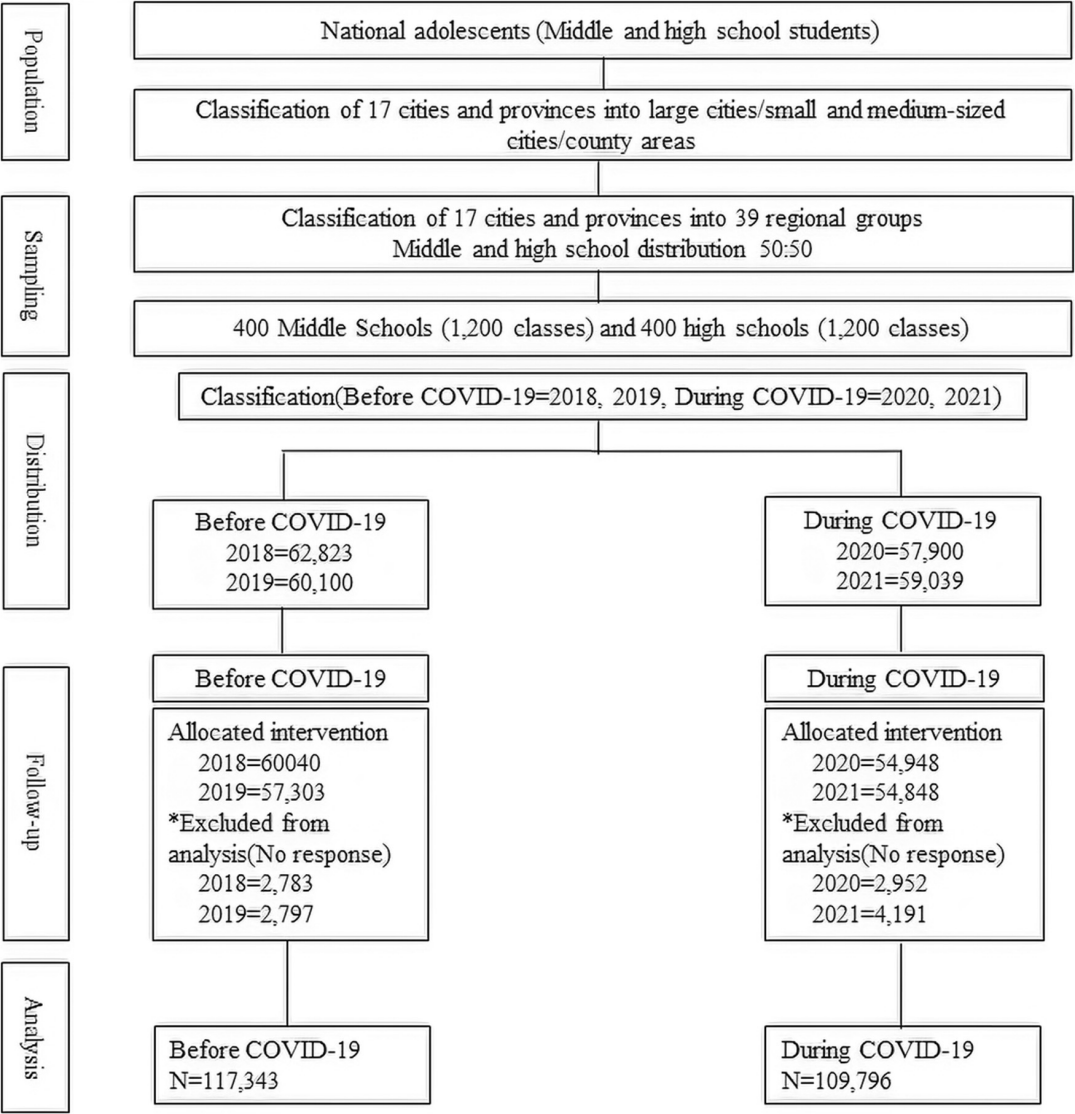
Flow diagram.

KCDC designated middle and high-school students across the nation as their research demographic. The administrative divisions included 39 regional clusters across 17 cities and provinces, with an equal allocation of middle and high schools in each region, maintaining a 50:50 ratio. Subsequently, schools from each city or province were sampled, encompassing 2,400 classes across 800 schools: 1,200 classes from 400 middle schools and 1,200 classes from 400 high schools, one class per grade. All students enrolled in these classes were included in the survey. KCDC also facilitated survey-training sessions for the respective teachers, who were then responsible for administering the surveys to their students (Figure 1).

By comparing data from 2018 and 2019 (before the pandemic) with data from 2020 and 2021 (during the pandemic), we examined PA and mental health among adolescents. After excluding missing values for each year, the final dataset included 60,040 responses from 2018, 57,303 responses from 2019, 54,948 responses from 2020, and 54,848 responses from 2021. The total number of responses before the pandemic was 117,343, and total number of responses during the pandemic was 109,796. The data were approved for use by KCDC’s Adolescent Health Behavior Survey (National Statistics No. 117058), which is publicly available and not subject to review by the Institutional Review Board. The data were collected through an anonymous self-administered online survey of students and includes essential health indicators for understanding the current health status of Korean adolescents.

The subject of this study includes both middle and high-school students in Korea. Middle-school students typically range in age from 12 to 15, entering school at about 12–13 years old and finishing at 15–16 years old. In contrast, high-school students begin their education at approximately 15–16 years old and graduate at 18–19 years old. The sample for the period before the COVID-19 pandemic consisted of 60,054 middle-school students and 57,289 high-school students. During the pandemic, the sample included 59,315 middle-school students and 50,481 high-school students. The average age of middle-school students was 13.62 years (SD = 0.957), and the average age of high-school students was 16.60 years (SD = 0.948).

### 2.2 Measures

The questionnaires we used for PA, stress, depression, and suicidal ideation were validated through previous research and confirmed in the annual survey conducted by KCDC in 2021. Reliability was further reinforced through their application up to the 17th survey. KCDC used various response standards for survey items. Consequently, we were not able to use uniform measurement units.

Demographic characteristics included gender, age, height, and weight. Age, height, and weight were collected through direct input from respondents. PA was measured by the number of days of moderate and high-intensity exercise, as well as frequency of strength training, within the previous 7 days. Moderate-intensity PA involved elevated heart rate or shortness of breath for more than 60 min, regardless of the type of exercise. This 8-point scale ranged from “not in the last 7 days” (1 point) to “7 days a week” (8 points). High-intensity PA involved shortness of breath or body sweating for more than 20 min (e.g., including jogging, soccer, basketball, taekwondo, mountain climbing, brisk cycling, brisk swimming, and heavy lifting. This 6-point scale ranged from “not in the last 7 days” (1 point) to “more than 5 days a week” (6 points). Strength training involved muscle-strengthening exercises, such as push-ups, sit-ups, weight lifting, dumbbells, iron bars, and parallel bars. This 6-point scale ranged from “not in the last 7 days” (1 point) to “at least 5 days a week” (6 points).

To analyze the difference in mental health according to regular moderate and high-intensity PA and strength training, we defined participants who engaged in PA less than once a week as non-participants and those who participated more than twice a week as regular participants. This criterion aligns with the standards used by the Korean government’s National Sport for All Participation Survey.

Mental health consisted of three variables: stress, depression, and suicidal ideation. Stress refers to the level of stress felt in everyday life, typically measured on a 5-point scale from “not felt at all” (1 point) to “very much felt” (5 points). In the current study, categorized responses such as “I don’t feel stress at all” and “I feel any stress” as “I don’t feel stress” (0 points) and responses such as “I feel a little stress,” “I feel a lot,” and “I feel a lot of stress” as “I feel stress” (1 point). The measure for incidence of depression and suicidal ideation was a dichotomous scale (0 = absence, 1 = presence). To ensure consistency across variables measuring mental health conditions and to enhance comparability, we use the same approach to measure stress. Depression was assessed based on whether respondents experienced sadness or despair to the extent that it disrupted their daily life for 2 weeks over the previous 12 months. Options were “not in the past 12 months” (0 points) and “present in the past 12 months” (1 point). Suicidal ideation refers to the presence of serious suicidal thoughts in the past 12 months. Options were “not in the past 12 months” (0 points) and “present in the past 12 months” (1 point). The higher the score in a given period, the higher the perceived level of stress, depression, and suicidal ideation.

### 2.3 Statistical analysis

The data were analyzed using the chi-square (*χ* ^2^) test and correlation analysis with SPSSWIN software (version 24.0; IBM Co., Armonk, NY, USA). Non-responses were excluded from analysis. Specific details are marked with asterisks in Figure 1. To analyze variations between two distinct timeframes, we divided the population (*N* = 239,862) into two groups: before COVID-19 (2018 and 2019) and during COVID-19 (2020 and 2021). Before COVID-19, the population numbers were 62,823 in 2018 and 60,100 in 2019. During COVID-19, the numbers were 57,900 in 2020 and 59,039 in 2021. During the follow-up phase, after adjusting for non-responses, the sample sizes were 60,040 in 2018, 57,303 in 2019, 54,948 in 2020, and 54,848 in 2021. In the final stage of analysis, we used data from 117,343 individuals before COVID-19 and 109,796 individuals during COVID-19 (Figure 1).

The chi-square test was used to examine changes in PA and mental health including stress, depression, and suicidal ideation among adolescents before and during the pandemic. Furthermore, correlation analysis was used to explore the relationship between regular PA and mental health in adolescents. Typically, if the absolute value of the correlation coefficient falls between 0.20 and 0.39, it indicates weak correlation. A value between 0.40 and 0.59 indicates moderate correlation, and a value above 0.60 indicates strong correlation. Since statistical significance level of 5% (0.05) is commonly used, this study also adheres to this standard.

Cramer’s V coefficient was used to assess the effect size (ES) of the chi-square test results for independence. Cramer’s V serves as a measure of the ES for the chi-square test, indicating the strength of the relationship between variables. When ES is less than or equal to 0.2, it suggests weak results. This means that even though the results are statistically significant, the fields are only weakly correlated. When ES falls within the range of 0.2–0.6, it is considered acceptable, indicating that the fields are appropriately related. ES greater than 0.6 signifies robust results, indicating a strong relationship between the fields. The goal in using non-parametric statistics (i.e., Cramer’s V) was to accommodate the data while ensuring accuracy and robustness in our analysis.

## 3 Results

### 3.1 Changes in PA before and during the pandemic

**Figure 2:**
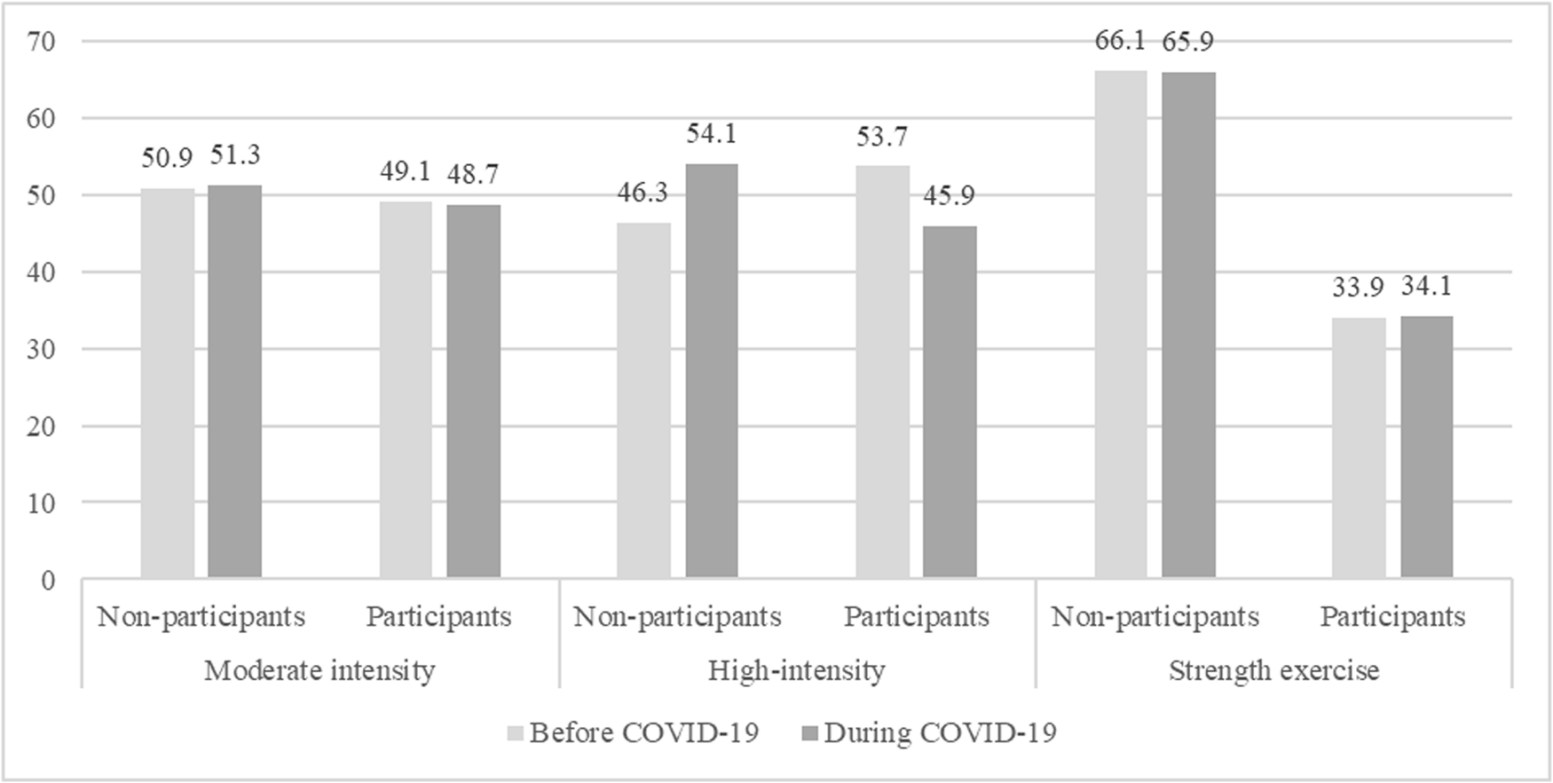
Changes in PA before and during COVID-19.

PA levels underwent noticeable changes from the period before the pandemic to the period during the pandemic (Figure 2). The statistically significant results are as follows. High-intensity PA participation experienced a significant decrease from 53.7% (*n* = 63,036) to 45.9% (*n* = 50,383), and non-participation increased significantly from 46.3% (*n* = 54,307) to 54.1% (*n* = 59,413) (*p* < 0.05, ES = 0.078).

### 3.2 Changes in stress before and during the pandemic

Both participants and non-participants in moderate-intensity PA experienced a decrease in stress, with participants showing consistently lower stress than non-participants (*p* < 0.05, ES = 0.053). Specifically, stress among participants decreased from 38.8 to 38.0%, while stress among non-participants decreased from 42.3 to 41.7% (Table 1). Participants in high-intensity PA experienced higher stress before the pandemic than non-participants (*p* < 0.05, ES = 0.080), but during the pandemic, their stress became lower than non-participants (*p* < 0.05, ES = 0.008). When comparing change in stress before and during the pandemic, participants in high-intensity PA decreased from 42.0 to 35.5%, while non-participants increased from 39.1 to 44.2%. Notably, before the pandemic, participants were more likely to experience stress than non-participants, but this trend reversed during the pandemic. Prior to the pandemic, participants in strength training were significantly less likely to experience stress than non-participants (*p* < 0.05, ES = 0.082); this pattern continued during the pandemic. Both groups showed a decrease in stress, from 26.0 to 25.8% for participants and from 55.1 to 53.9% for non-participants.

**Table 1:** Changes in stress by PA before and during COVID-19

		Before COVID-19		During COVID-19		*p*-value
Variable		Stress non-experience	Stress experience	Stress non-experience	Stress experience	
Moderate-intensity	Non-participants	10,093 (8.6%)	49,620 (42.3%)	10,489 (9.6%)	45,785 (41.7%)	0.000
	Participants	12,119 (10.3%)	45,511 (38.8%)	11,795 (10.7%)	41,727 (38.0%)	0.000
High-intensity	Non-participants	8,454 (7.2%)	45,853 (39.1%)	10,833 (9.9%)	48,580 (44.2%)	0.000
	Participants	13,758 (11.7%)	49,278 (42.0%)	11,451 (10.4%)	38,932 (35.5%)	0.005
Strength training	Non-participants	12,905 (11.0%)	64,647 (55.1%)	13,175 (12.0%)	59,175 (53.9%)	0.000
	Participants	9,307 (7.9%)	30,484 (26.0%)	9,109 (8.3%)	28,337 (25.8%)	0.304
Total		22,212 (18.9%)	95,131 (81.1%)	22,284 (20.3%)	87,512 (79.7%)	

### 3.3 Changes in depression before and during the pandemic

Before the pandemic, participants who engaged in moderate-intensity PA were less likely to experience depression than non-participants (*p* < 0.05, ES = 0.007), and this trend continued during the pandemic (*p* < 0.05, ES = 0.013). Both participants and non-participants in moderate-intensity PA demonstrated a decrease in depression during the pandemic (Table 2). Before the pandemic, participants engaging in high-intensity PA had a higher rate of depression than non-participants (*p* < 0.05, ES = 0.022), but during the pandemic, their rate of depression was lower than non-participants (*p* < 0.05, ES = 0.008). Specifically, depression decreased from 14.3 to 12.1% in high-intensity PA participants and slightly increased from 13.2 to 13.9% in non-participants. Participants who engaged in strength training before and after the pandemic had significantly lower rates of depression than those who did not engage in strength training (*p* < 0.05, ES = 0.010). Depression among strength training participants decreased from 9.1 to 8.8%, while among non-participants, it decreased from 18.4 to 17.2%. These findings provide evidence that regular strength training can have a positive impact on mental health, even during a pandemic.

**Table 2:** Changes in depression by PA before and during COVID-19

		Before COVID-19		During COVID-19		*p*-value
Variable		Depression non-experience	Depression experience	Depression non-experience	Depression experience	
Moderate-intensity	Non-participants	43,129 (36.8%)	16,584 (14.1%)	41,959 (38.2%)	14,315 (13.0%)	0.019
	Participants	41,978 (35.8%)	15,652 (13.3%)	39,305 (35.8%)	14,217 (12.9%)	0.000
High-intensity	Non-participants	38,826 (33.1%)	15,481 (13.2%)	44,176 (40.2%)	15,237 (13.9%)	0.000
	Participants	46,281 (39.4%)	16,755 (14.3%)	37,088 (33.8%)	13,295 (12.1%)	0.005
Strength training	Non-participants	56,001 (47.7%)	21,551 (18.4%)	53,478 (48.7%)	18,872 (17.2%)	0.001
	Participants	29,106 (24.8%)	10,685 (9.1%)	27,786 (25.3%)	9,660 (8.8%)	0.304
Total		85,107 (72.5%)	32,236 (27.5%)	81,264 (74.0%)	28,532 (26.0%)	

### 3.4 Changes in suicidal ideation before and during the pandemic

Suicidal ideation among moderate-intensity PA participants was significantly lower before and during the pandemic than non-participants (*p* < 0.05, ES = before 0.021, during 0.012). During the pandemic, the rate of suicidal ideation decreased from 6.1 to 5.6% for participants and from 7.1 to 6.2% for non-participants (Table 3). High-intensity PA participants had the same rate of suicidal ideation as non-participants before the pandemic at 6.6% (*p* < 0.05, ES = 0.031). However, during the pandemic, the rate of not experiencing suicidal ideation was higher for participants than non-participants (*p* < 0.05, ES = 0.014). During the pandemic, suicidal ideation decreased from 6.6 to 5.2% among participants and remained unchanged at 6.6% among non-participants. For strength training participants, suicidal ideation was more than half that of non-participants before and during the pandemic (*p* < 0.05, ES = before 0.022, during 0.022). During the pandemic, participants decreased from 4.1 to 3.7%, and non-participants decreased from 9.1 to 8.1%. A notable result is that overall, the rate of suicidal ideation decreased for all types of PA during the pandemic.

**Table 3:** Changes in suicidal ideation by PA before and during COVID-19

		Before COVID-19		During COVID-19		*p*-value
Variable		Suicidal ideation non-experience	Suicidal ideation experience	Suicidal ideation non-experience	Suicidal ideation experience	
Moderate-intensity	Non-participants	51,419 (43.8%)	8,294 (7.1%)	49,436 (45.0%)	6,838 (6.2%)	0.000
	Participants	50,450 (43.0%)	7,180 (6.1%)	47,425 (43.2%)	6,097 (5.6%)	0.000
High-intensity	Non-participants	46,531 (39.7%)	7,776 (6.6%)	52,170 (47.5%)	7,243 (6.6%)	0.000
	Participants	55,338 (47.2%)	7,698 (6.6%)	44,691 (40.7%)	5,692 (5.2%)	0.000
Strength exercise	Non-participants	66,909 (57.0%)	10,643 (9.1%)	63,449 (57.8%)	8,901 (8.1%)	0.000
	Participants	34,960 (29.8%)	4,831 (4.1%)	33,412 (30.4%)	4,034 (3.7%)	0.000
Total		101,869 (86.8%)	15,474 (13.2%)	96,861 (88.2%)	12,935 (11.8%)	

### 3.5 Correlation among the pandemic, age, PA, and mental health

We made the pandemic a dummy variable, assigning a score of 0 to “before the pandemic” and a score of 1 to “during the pandemic,” for correlation analysis with other variables.

The findings indicate a negative correlation between the pandemic and high-intensity PA (*r* = −0.078), stress (*r* = −0.017), depression (*r* = −0.017), and suicidal ideation (*r* = −0.021). These findings suggest that during the pandemic, high-intensity PA, stress, depression, and suicidal ideation decreased. Although the correlation coefficient seems small, this trend is notable.

Age showed a negative correlation with moderate intensity PA (*r* = −0.084), high-intensity PA (*r* = −0.116), and strength training (*r* = −0.041). Conversely, age showed a positive correlation with stress (*r* = 0.068) and depression (*r* = 0.046) but a negative correlation with suicidal ideation (*r* = −0.004). These results suggest that a decrease in exercise frequency and suicidal ideation with increasing age might relate to increases in stress and depression (Table 4).

**Table 4:** Correlation analysis on COVID-19, PA, and mental health

	1	2	3	4	5	6	7	8
1. COVID-19	1							
2. Age	0.0290^***^	1.000						
3. Moderate-intensity	−0.004	−0.084^***^	1.000					
4. High-intensity	−0.078^***^	−0.116^***^	0.522^***^	1.000				
5. Strength training	0.002	−0.041^***^	0.353^***^	0.436^***^	1.000			
6. Stress	−0.017^***^	0.068^***^	−0.047^***^	−0.066^***^	−0.077^***^	1.000		
7. Depression	−0.017^***^	0.046^***^	0.003	−0.006^**^	−0.007^**^	0.224^***^	1.000	
8. Suicidal ideation	−0.021^***^	−0.004^*^	−0.017^***^	−0.021^***^	−0.022^***^	0.156^**^	0.402^***^	1.000

COVID-19 was made variable by assigning 0 points for before COVID-19 and 1 point for during COVID-19; **p < 0.01 ***p < 0.001.

Moderate-intensity PA had a weak negative correlation with stress (*r* = −0.047) and suicidal ideation (*r* = −0.017), while high-intensity PA had negative correlations with stress (*r* = −0.066), depression (*r* = −0.006), and suicidal ideation (*r* = −0.021). Similarly, strength training had a negative correlation with stress (*r* = −0.077), depression (*r* = −0.007), and suicidal ideation (*r* = −0.022).

Significantly, a positive correlation emerged among the mental health sub-variables, with a relatively high correlation (*r* = 0.402) between depression and suicidal ideation. These results suggest that regular PA is directly related to various mental health variables and could reduce suicidal ideation by relieving stress and depression. Overall, the findings highlight the potential benefits of regular PA for mental health, especially during a pandemic.

## 4 Discussion

We examined the association between moderate-intensity PA and mental health (i.e., stress, depression, and suicidal ideation) in Korean adolescents before and during the COVID-19 pandemic. To this end, we used nationwide data obtained from KCDC. Leveraging nationwide data rectified, to some extent, the shortcomings of previous studies and provided a valuable reference point for investigating similar situations in the future. The findings also provide insights for developing effective interventions and strategies to meet the needs of adolescents.

### 4.1 PA level before and during pandemic

Levels of moderate- and high-intensity PA were significantly different before and during the pandemic, supporting H1, which proposed that PA level before and during the COVID-19 pandemic would differ. Among non-participants in high-intensity PA before the pandemic, high-intensity PA level increased by 7.8% during the pandemic, a change influenced by government policies and recommendations [33], as well as additional leisure time resulting from remote schooling [34]. Previous findings confirm the same [35]. Among non-participants in moderate-intensity PA before the pandemic, moderate-intensity PA level increased by 0.4% during the pandemic, while decreasing by 0.4% among participants. Previous findings indicate the same among university students in Australia, Croatia, England, Hungary, Italy, Mexico, Spain, and the United States [36–38]. On the one hand, PA level among moderate-intensity PA participants slightly decreased during the pandemic, likely due to restrictions such as home quarantine and suspension of operation of sports venues [4–7,36–38]. On the other hand, PA level among non-participants in moderate-intensity PA slightly increased during the pandemic, likely because isolation at home made people crave health and regular moderate-intensity exercise [34,36–38].

Notably, among participants in high-intensity PA before the pandemic, high-intensity PA level decreased by 7.8% during the pandemic. In extensive descriptive studies involving nearly 500,000 participants from the United Kingdom, Ireland, New Zealand, and Australia during the initial stages of COVID-19 containment by national governments, individuals with pre-existing exercise habits experienced a decrease in PA level [39,40]. This observation suggests that during national efforts to contain COVID-19, individuals who did not normally engage in PA found opportunities to change their exercise behaviors, potentially leading to long-term health benefits. Meanwhile, our findings also underscore the importance of reinforcing public health policies to encourage Korean youth to be more active in sports, especially during and after a pandemic such as influenza [33].

### 4.2 Stress by PA level

Given different intensities of PA, participants had significantly different stress levels before and during the pandemic, supporting H2, which proposed that adolescents participating in moderate- and high-intensity PA and strength training would experience lower stress levels than those who did not participate in such activities. This result suggests the impact of PA level on stress among Korean adolescents before and during the pandemic. Notably, we found that adolescents who participated in moderate-intensity PA consistently exhibited lower stress than those who did not engage in such activities. This pivotal finding indicates that PA can positively influence mental health among adolescents.

Moreover, adolescents engaging in high-intensity PA experienced higher stress levels before the pandemic than during the pandemic and experienced lower stress during the pandemic than non-participants. These shifts could be due to the impact of alterations in daily routine (e.g., transition to online classes and school closures) on both PA level and stress. Previous findings demonstrate that PA can have a positive impact on mental health [20,21], with higher levels of PA associated with lower stress related to COVID-19 [22]. Regarding the relationship between PA and stress, behaviors such as reducing academic and social burdens, enhancing family coherence, and promoting health seeking could mediate the alleviation of severe stress and suicidality in youths [41]. Furthermore, engaging in sufficient and regular PA can release psychological tension and enhance mental stability. Previous findings highlight the detrimental mental health effects associated with low PA, including higher stress [29,42–44].

### 4.3 Depression by PA level

Given different intensities of PA, participants had significantly different depression rates before and during the pandemic, supporting H3, which proposed that adolescents participating in moderate- and high-intensity PA and strength training would experience lower depression levels than those who did not participate in such activities. Exercise and PA have beneficial effects that are often comparable to antidepressant treatment [45,46]. Before the pandemic, rates of depression were lower in both participants and non-participants in moderate-intensity PA, and high-intensity PA participants had lower rates of depression during the pandemic than non-participants. The intensity of PA not significantly affects depression rates under typical conditions. However, participants in high-intensity PA during the pandemic had lower rates of depression than non-participants, suggesting that during stressful conditions such as a pandemic, intensity of PA might have a more pronounced impact on mental health [47], raising the possibility that high-intensity PA might be more effective in preventing depression. High-intensity interval training and moderate-intensity training can reduce stress, anxiety, and depression and improve recovery, but improvement in depression in the high-intensity interval trainers was greater than in the moderate-intensity trainers. Indeed, short recovery periods can alleviate the stress of intense exercise [48–51].

These insights clarify the relationship between PA and mental health, providing valuable guidance for developing health management strategies, especially in stressful environments. However, participants in high-intensity PA before the pandemic had higher rates of depression than non-participants, suggesting that an individual’s physical condition, frequency of exercise, and stress level all affect the effectiveness of PA. Participants in strength training before and after the pandemic had significantly lower rates of depression than non-participants, suggesting this form of exercise might prevent depression through mechanisms such as relieving stress, enhancing self-esteem, and improving overall physical health [52].

### 4.4 Suicidal ideation by PA level

Given different intensities of PA, participants had significantly different suicidal ideation before and during the pandemic, supporting H4, which proposed that adolescents participating in moderate- and high-intensity PA and strength training would experience lower suicidal ideation levels than those who did not participate in such activities. When faced with a highly contagious disease, people might experience intolerable psychological distress that triggers harmful behaviors [53]. These feelings might lead to anxiety, depression, and poor mental health; in some cases, it can also lead to suicidal ideation [54,55]. Regular participation in PA is often the first step in preventing and managing chronic disease [56]. Regular PA can alleviate the impact of depressive symptoms resulting from the psychological burden of the COVID-19 situation [47]. PA is an important factor in managing mental and physical health [57], and regular participation in PA can reduce the risk of suicide-related outcomes [58], increasing mental stability during a pandemic.

Our findings show a decrease in the incidence of suicidal ideation across all types of PA [27]. Participants who engaged in moderate-intensity PA before and during the pandemic had significantly lower suicidal ideation than non-participants, suggesting that PA can have a positive impact on mental health during stressful situations. Notably, rates of suicidal ideation decreased during the pandemic among participants, while rates remained the same among non-participants. This finding highlights the role of PA in maintaining mental health during stressful times, perhaps because participation in PA can cultivate positive emotions that are often lacking in people with high levels of depression [59]. Mental health was an important preventive factor for suicidal ideation during the pandemic, as low levels of positive mental health relate to high levels of depressive symptoms [60]. Our findings suggest that PA might reduce suicidal ideation by reducing stress responses, enhancing self-esteem, and improving overall mental health. Additionally, the findings suggest that PA can manage risk of depression and suicide, particularly in stressful situations.

During the pandemic, rates of suicidal ideation decreased significantly among PA participants, while rates remained stable among non-participants. Although these results suggest an association between PA participation and lower suicidal ideation, determining causality remains challenging. In other words, scholars should consider randomized controlled trials to determine whether this relationship is causal. Nonetheless, PA has positive effects such as reducing stress, improving mood, and alleviating anxiety, all of which might help prevent suicidal thoughts. Furthermore, PA can induce physiological changes that positively influence mental health. Those who did not participate in strength training, before and during the pandemic, had rates of suicidal ideation more than twice as high as those who did participate in strength training. However, both groups experienced lower suicidal ideation during the pandemic. The observed association between strength training (a form of PA) and suicidal ideation requires further investigation to determine whether a causal relationship exists. In addition, exploring the links between various types and intensities of exercise and factors such as mental health status, social support, and economic status, and suicidal ideation would be worthwhile. At the same time, the significant decline in suicidal ideation in PA participants and non-participants during the pandemic is worthy of attention. This observed decline might be attributable to higher social, economic, and psychological support or to individual adaptation to the pandemic.

We found an overall negative correlation between moderate-intensity PA, high-intensity PA, and strength training and mental health indicators such as stress, depression, and suicidal ideation. These findings suggest that PA can significantly contribute to reductions in stress, depression, and suicidal ideation, thereby enhancing overall mental health. Furthermore, the observed efficacy of moderate and vigorous exercise in reducing stress implies that all forms of PA are beneficial; therefore, individuals can choose PA based on personal preference and circumstance. The most significant correlation observed between PA and stress suggests that PA has the potential to reduce stress hormones, enhance positive mood, and relieve stress symptoms by inducing changes in neurotransmitters in the brain. These findings highlight the critical role that various forms of PA can play in reducing stress, pointing to the development of preventive health management strategies.

### 4.5 Relationships among pandemic, age, PA level, and mental health

Our findings support H5, revealing associations among pandemic, age, PA, and mental health in adolescents. Pandemic had negative relationships with moderate- and high-intensity PA and mental health (e.g., stress, depression, and suicidal ideation). These results suggest that the pandemic reduced PA, primarily due to social distancing and restricted access to or closures of sports facilities. The paradoxical positive relationship between the pandemic and mental health improvement is attributable to Korea’s special situation. Korean adolescents tend to experience academic stress due to the demands of studying and private academy attendance after regular school hours. The pandemic reduced this burden by forcing schools and private academies to move online. Previous findings confirm that the transition to online courses and the closure of schools and universities during the pandemic alleviated academic burdens on Korean adolescents and improved their mental health [16–19,41].

In the case of Korea, PA decreased as adolescents needed to manage more academic work in preparation for college entrance exams as they reach higher grades. Although only to a small degree, suicidal ideation decreased with age and maturity, suggesting that the ability to cope improved. However, Korean adolescents still experienced higher levels of stress and depression as they aged. This increase might relate to changes in environment and identity [61], suggesting the need to develop mental health interventions and support systems appropriate for adolescents.

Additionally, PA level had a positive correlation with overall mental health in terms of stress, depression, and suicidal ideation. This relationship reveals the value and importance of PA for mental health. Regular PA during adolescence could be a meaningful mental health intervention. Kim et al. found that PA lowered stress and depression in adolescents [20]. During a pandemic, PA at all levels could be an effective way to improve adolescent mental health [21–24].

Considering the above results and discussions, we obtain the following implications. COVID-19 is a virus that we needed to fight for a long time. In order to promote better mental health among adolescents, the government and health departments need to formulate effective and reasonable epidemic prevention policies, facilitate health and fitness based on the growth characteristics of adolescents, advocate for sports activities, develop reasonable PA and mental health training prescriptions, actively encourage adolescents to participate in PA, help adolescents maintain mental health, reduce the prevalence of mental diseases, and improve awareness of healthy behaviors among adolescents. The findings of this study also provide a valuable reference point for managing similar challenging situations in the future.

## 5 Conclusion

The objective of this study was to analyze changes in PA and mental health among Korean adolescents before and during the COVID-19 pandemic. Based on data from the KCDC Adolescent Health Behavior Survey, our findings indicate significant trends. A modest decline in moderate-intensity PA and a significant rise in non-participation emerged during the pandemic. A notable decrease in high-intensity PA and a marked increase in non-participation were also evident. However, participation in strength training slightly increased, with only a minor decrease in non-participation.

Adolescents who participated in moderate-to-high-intensity PA reported less frequent stress than non-participants, marking a shift from before the pandemic. Although stress among strength training participants was not conclusively lower, those who were active before the pandemic experienced a notable reduction. Similarly, adolescents involved in moderate-to-high-intensity PA experienced fewer depressive episodes both before and during the pandemic. Interestingly, before the pandemic, high-intensity PA participants reported higher rates of depression than non-participants, but this trend reversed during the pandemic. Individuals who participated in strength training before the pandemic showed significantly lower rates of depression during the pandemic, suggesting that regular strength training might prevent depression.

Furthermore, suicidal ideation decreased among moderate-intensity PA participants both before and during the pandemic. In contrast, while high-intensity PA participants initially showed rates similar to non-participants, their rates significantly dropped during the pandemic, unlike those of non-participants. Participation in strength training consistently correlated with lower suicide ideation.

Pandemic is negatively related to moderate- and high-intensity PA and mental health (e.g., stress, depression, and suicidal ideation). As age increased, PA and suicidal ideations decreased, while stress and depression increased. Overall, mental health tended to improve as adolescents participated in PA.

Developing and implementing tailored guidelines for PA and strength training is crucial to adolescent well-being, especially during a crisis like a pandemic. Encouraging moderate-intensity PA is vital due to its pandemic-induced decline, and efforts to counteract the increase in non-participation are necessary. Accessible, engaging opportunities for moderate-intensity PA within schools and communities are essential. Moreover, promoting diverse PA, including alternatives such as strength training, and highlighting the mental health benefits of PA in educational and community settings are potentially helpful. Developing and disseminating evidence-based PA and strength-training guidelines, tailored to the needs and preferences of adolescents, are paramount. A holistic approach, integrating social and family support, is likely to enhance the well-being of young people.

The current study has two important limitations. First, while the findings provide valuable insights into Korean adolescents, cultural factors influencing PA participation and mental health perceptions might not translate directly to adolescents in other cultural contexts. In this way, the findings have limited generalizability. Second, our comparative cross-sectional design prevented comparison of the same subjects before and during the pandemic. For this reason, the findings might reflect variations between different groups of subjects rather than longitudinal evolution of individual behaviors and mental health states. In future studies, scholars should seek data that more reliably permits longitudinal analysis.
